# Endoscopic ultrasound‐guided tissue acquisition for focal liver lesions in patients with a history of multiple primary malignant neoplasms

**DOI:** 10.1002/deo2.372

**Published:** 2024-04-30

**Authors:** Yuichi Takano, Naoki Tamai, Masataka Yamawaki, Jun Noda, Tetsushi Azami, Fumitaka Niiya, Fumiya Nishimoto, Naotaka Maruoka, Tatsuya Yamagami, Masatsugu Nagahama

**Affiliations:** ^1^ Department of Internal Medicine, Division of Gastroenterology Showa University Fujigaoka Hospital Kanagawa Japan

**Keywords:** endoscopic ultrasound, focal liver lesion, hepatocellular carcinoma, metastatic liver tumor, tissue acquisition

## Abstract

**Objective:**

This study aimed to investigate the usefulness of endoscopic ultrasound‐guided tissue acquisition (EUS‐TA) for diagnosing focal liver lesions in patients with a history of multiple primary malignant neoplasms.

**Methods:**

Among patients who underwent EUS‐TA for focal liver lesions between 2016 and 2022, those with a history of multiple malignant neoplasms were included. A histologically confirmed malignant tumor within the past 5 years before EUS‐TA was defined as a history of malignant neoplasm. The primary outcomes were diagnostic ability and adverse events of EUS‐TA.

**Results:**

This study included 16 patients (median age, 73 [33–90] years), the median tumor size was 32 (6–51) mm, 14 had a history of double malignant neoplasms, whereas two had triple malignant neoplasms. Malignant neoplasms were detected histologically or cytologically in all cases. Immunohistochemistry was performed in 75% (12/16), and the final diagnosis of EUS‐TA was metastatic liver tumor in 12 patients, and primary malignant liver tumor in four patients. The primary site could be identified in 11 of 12 metastatic tumor cases. The diagnostic yield of EUS‐TA was 100% (16/16) for differentiating benign and malignant tumors and 94% (15/16) for confirming the histological type including the primary site of metastatic lesions. No adverse events were associated with the procedure.

**Conclusion:**

EUS‐TA is a useful diagnostic modality for focal liver lesions in patients with a history of multiple malignant neoplasms, allowing for the differential diagnosis of primary and metastatic tumors and identification of the primary site of metastatic lesions.

## INTRODUCTION

With the remarkable development of imaging modalities, most focal liver lesions can be diagnosed without histological examination.^1^, ^2^ However, in patients with a history of multiple malignant neoplasms, focal liver lesion diagnosis can be complicated. The differential diagnosis includes primary malignant liver tumors (hepatocellular carcinoma and intrahepatic cholangiocarcinoma), metastatic liver tumors, and benign lesions, and histological evaluation is essential for definitive diagnosis. In metastatic liver tumors, the treatment strategy differs depending on the primary site; therefore, identifying the primary site is important. Traditionally, ultrasound (US)‐guided or computed tomography‐guided percutaneous liver biopsy was the gold standard tissue sampling method for focal liver lesions,^3^, ^4^ but reports of endoscopic US‐guided tissue acquisition (EUS‐TA) have increased recently.^5^ However, the usefulness of EUS‐TA in diagnosing focal liver lesions associated with multiple malignant neoplasms has not been reported.

### Aim

In this study, we investigated the usefulness of EUS‐TA for focal liver lesions in patients with a history of multiple malignant neoplasms.

### Method

This single‐center, retrospective case series study was approved by the ethics committee of Showa University. The inclusion criteria were patients with a history of multiple primary malignant neoplasms who underwent EUS‐TA for local liver lesions between 2016 and 2022. The exclusion criteria were as follows: (1) cases in which puncture was impossible due to intervening blood vessels; (2) uncorrectable bleeding tendency. The primary outcomes were diagnostic ability and adverse events of EUS‐TA.

### Definition

A histologically confirmed primary malignant tumor within the past 5 years before EUS‐TA was defined as a history of malignant neoplasm. Cytologically or histologically confirmed malignant results indicated malignancy, whereas histologically confirmed benign results with no tumor growth after 1‐year follow‐up indicated a benign tumor. A diagnosis of histological type was defined as a successful diagnosis of tumor histological type (for metastatic liver tumors, successful diagnosis of the primary site). The definition of adverse events established by the workshop of the American Society of Gastrointestinal Endoscopy was used.^6^

### EUS‐TA procedure

In EUS‐TA, analgesics and sedatives (pethidine hydrochloride [35 mg] or pentazocine [7.5–15 mg] + midazolam [1.5–4.5 mg]) were administered. Furthermore, a GF‐UCT260 endoscope (Olympus Medical Systems) and a UE‐ME1 or UE‐ME2 observation device (Olympus Medical Systems) were used. The puncture needle size was 22–25 G according to the operator's discretion. The number of strokes was 10–20, and the suction pressure was 10–20 ml negative pressure. Rapid on‐site evaluation of the cytology was not performed. Four endoscopists with experience of more than 50 cases performed the EUS‐TA. The needles used were Expect SlimLine Needle (Boston Scientific Japan) and SonoTip TopGain (Medico's Hirata). There were no cases in which contrast‐enhanced EUS was performed in this study.

### Pathological examination

Tissues obtained from EUS‐TA were fixed in formalin, followed by histological diagnosis using hematoxylin and eosin (HE) staining. Immunohistochemistry (IHC) was performed as necessary. After the tissue was fixed in formalin, the remaining liquid component was cytologically examined, with Papanicolaou staining. The cytological examination was employed as an auxiliary diagnosis.

## RESULTS

### Clinical background of the patients undergoing EUS‐TA

Out of 109 patients who underwent EUS‐TA for liver tumors between 2016 and 2022, 17 had a history of multiple malignant neoplasms. EUS‐TA was technically unsuccessful only in one case because of intervening vessels. This was the case of a man in his 80s with a history of sigmoid colon cancer and bladder cancer. EUS‐TA was attempted for a 26‐mm tumor in the right lobe of the liver (S5/8). Although the tumor could be identified from the duodenal bulb, EUS‐TA was not feasible because of intervening blood vessels. This case was excluded from the study.

The median age of 16 patients was 73 (33–90) years, with 11 males and five females. The median liver tumor size was 32 (6–51) mm. The tumor localized to the left lobe of the liver in 14 patients and the right lobe in 2 patients. Furthermore, 14 had a history of double primary malignant neoplasms, whereas two had triple malignant neoplasms. Primary tumors included colorectal cancer (eight cases), gastric cancer (four cases), pancreatic cancer (four cases), breast cancer (four cases), prostate cancer (three cases), esophageal cancer (two cases), renal cancer (two cases), bladder cancer (two cases), vocal cord cancer (one case), pancreatic neuroendocrine tumor (one case), lung cancer (one case), extrahepatic bile duct cancer (one case), and malignant lymphoma (one case), respectively (with overlaps). Antithrombotic drugs were used in three patients, and EUS‐TA was performed in all patients after drug discontinuation in accordance with the Japanese Gastrointestinal Endoscopy Society guidelines^7^ (Table 1).

**TABLE 1:** Clinical background of the patients undergoing endoscopic ultrasound‐guided tissue acquisition.

Number of patients	16
Age, median (range), years	73 (33–90)
Sex, male: female, no.	11:5
Size, median (range), mm	32 (6–51)
Location of the tumor,	
Left lober, no. (%)	14 (88)
Right lober, no. (%)	2 (12)
History of malignant neoplasms	
Double malignant neoplasms, no. (%)	14 (88)
Triple malignant neoplasms, no. (%)	2 (12)
Breakdown of malignant neoplasms (with overlaps)	
Colorectal cancer, no. (%)	8 (50)
Gastric cancer, no. (%)	4 (25)
Pancreatic cancer, no. (%)	4 (25)
Breast cancer, no. (%)	4 (25)
Prostata cancer, no. (%)	3 (18)
Esophageal cancer, no. (%)	2 (12)
Renal cancer, no. (%)	2 (12)
Bladder cancer, no. (%)	2 (12)
Vocal cords cancer, no. (%)	1 (6)
Pancreatic neuroendocrine tumor, no. (%)	1 (6)
Lung cancer, no. (%)	1 (6)
Extrahepatic bile duct cancer, no. (%)	1 (6)
Malignant lymphoma, no. (%)	1 (6)
Oral antithrombotic drug, no. (%)	3 (18)

### Outcomes of EUS‐TA

The puncture needles used were 22 G in 10 patients and 25 G in six patients. The needle types were Expect SlimLine and SonoTip TopGainTM in 11 and five patients, respectively.

Malignant neoplasms were histologically detected in 15 of 16 patients. One patient was diagnosed with inadequate material histologically but adenocarcinoma (Class V) cytologically.

**FIGURE 1:**
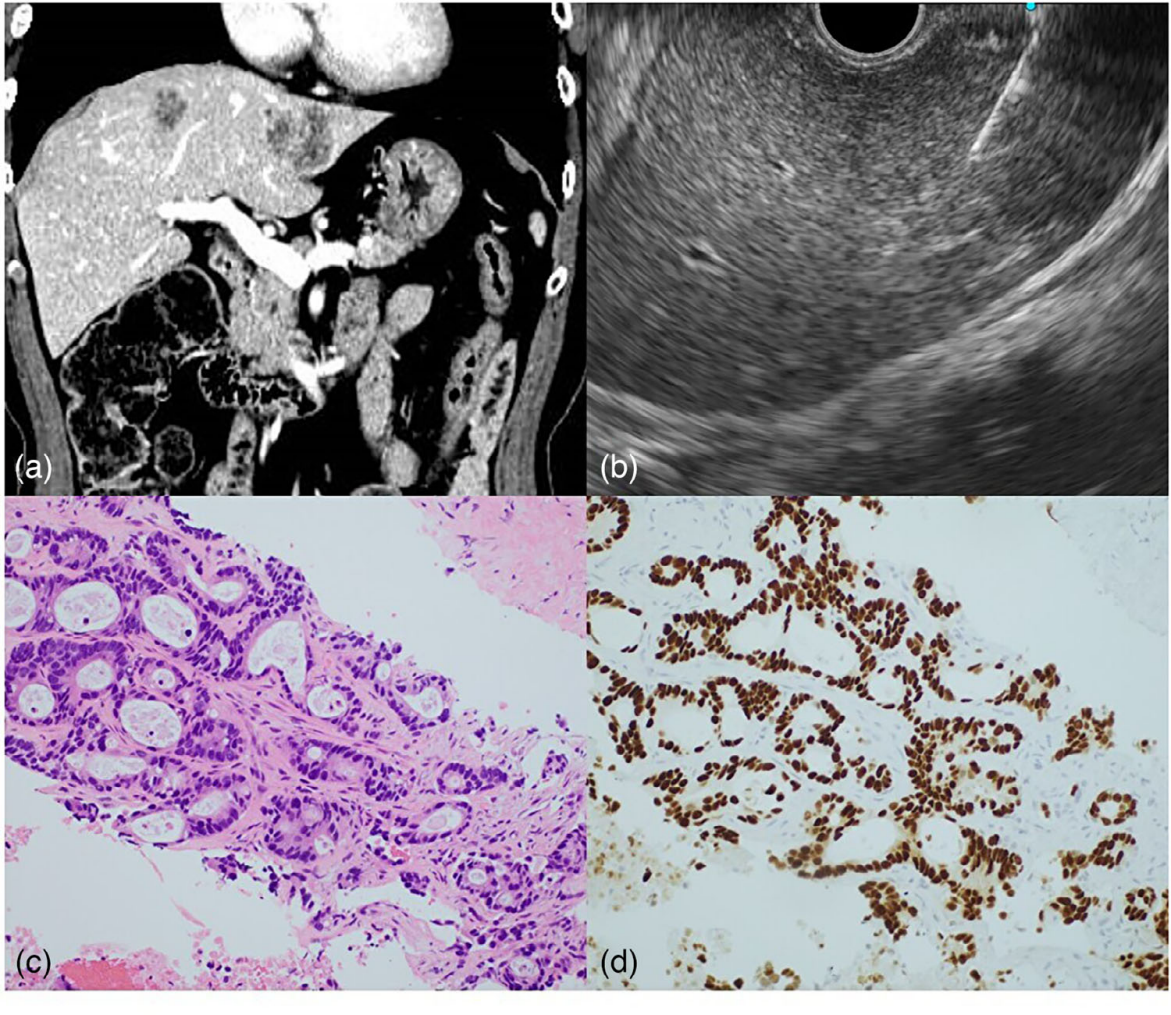
(a) A 77‐year‐old male with concurrent gastric cancer (poorly differentiated adenocarcinoma) and sigmoid colon cancer (well‐differentiated adenocarcinoma). Abdominal computed tomography showed multiple liver tumors in the left lobe, and metastasis from gastric or colorectal cancer was suspected. If the metastasis originated from gastric cancer, hepatic resection was contraindicated. If the metastasis originated from colorectal cancer, aggressive liver resection was recommended. (b) Endoscopic ultrasound‐guided tissue acquisition was performed transgastrically using a 22 G puncture needle. (c) Adenocarcinoma was histologically detected. (d) Immunohistochemistry showed positive for CDX‐2, making the diagnosis of metastasis from colorectal cancer possible. A left hepatectomy was performed. Pathologically, all liver lesions were metastases from colorectal cancer.

**FIGURE 2:**
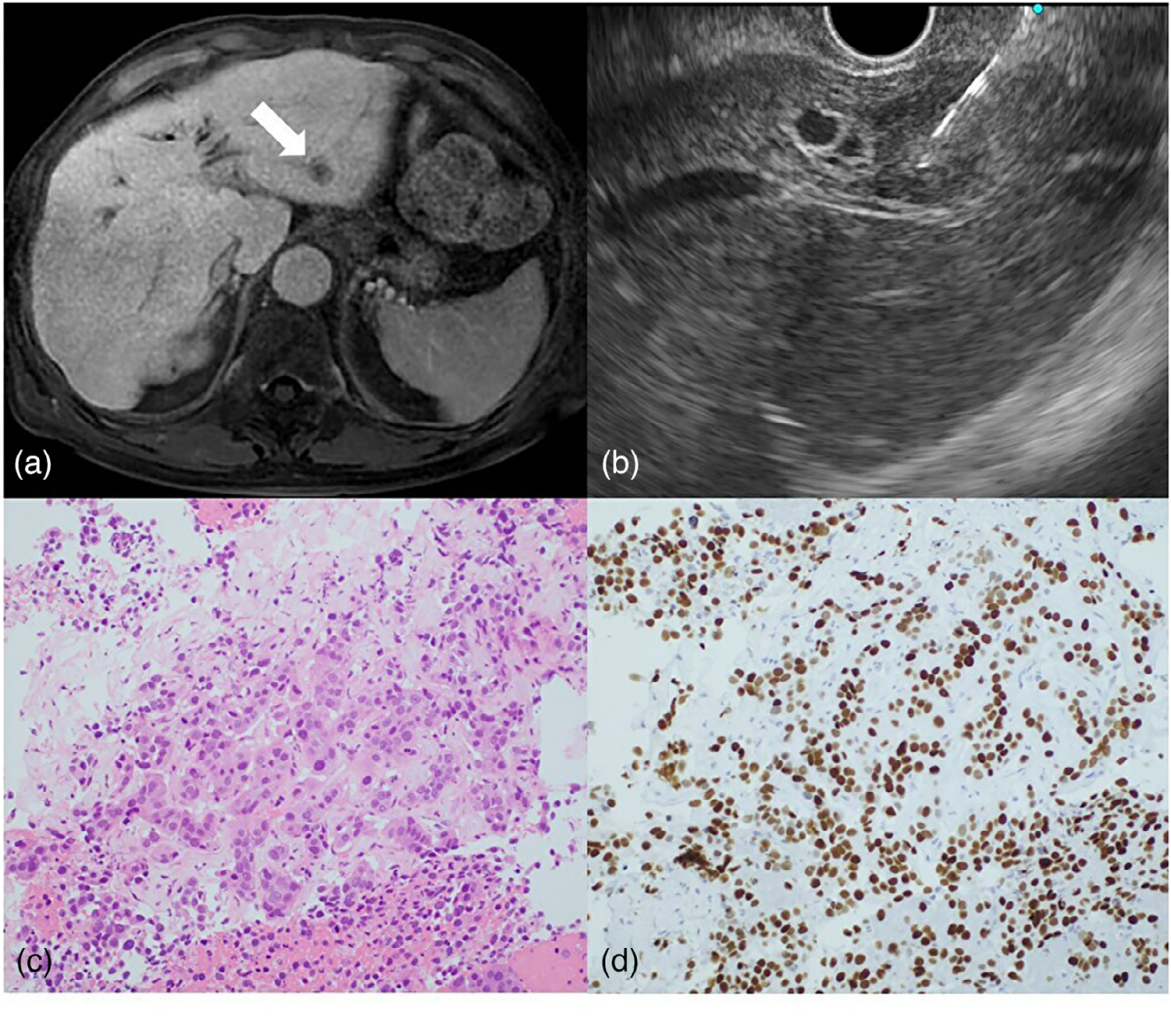
(a) An 81‐year‐old male with a history of triple malignant neoplasms (extrahepatic bile duct cancer, colorectal cancer, and lung cancer; all were pathologically diagnosed as adenocarcinoma). During follow‐up, contrast‐enhanced magnetic resonance imaging revealed a 15 mm tumor in the lateral area of the liver (arrow). (b) Endoscopic ultrasound‐guided tissue acquisition was performed transgastrically with a 22 G puncture needle. (c) Histologically, adenocarcinoma was observed. (d) Immunohistochemistry showed TTF‐1 positivity, and the patient was then diagnosed with lung cancer metastasis. Thus, chemotherapy for lung cancer was administered.

IHC was performed in 75% (12/16), and the final diagnosis was a metastatic liver tumor in 12 patients and a primary malignant liver tumor in four patients (two with hepatocellular carcinoma and two with intrahepatic cholangiocarcinoma). The primary site could be identified in 11 of 12 patients with metastatic liver tumors: five with colorectal cancer, three with pancreatic cancer, one with lung cancer, one with breast cancer, and one with pancreatic neuroendocrine tumor (Figures 1 and 2). On the basis of the clinical course, the remaining one case was diagnosed as a metastatic liver tumor derived from pancreatic cancer.

Based on the abovementioned statements, the EUS‐TA's diagnostic yield in differentiating between benign and malignant tumors was 100% (16/16), and its diagnostic ability in determining histological type was 94% (15/16). No procedure‐related adverse events were noted (Table 2). Based on the EUS‐TA diagnosis, four patients were judged to have indications for surgery, and they underwent surgery. In ten cases, the EUS‐TA diagnosis was useful in determining the chemotherapy regimen. In the remaining two cases, there was no impact on the treatment policy, as the best supportive care was chosen.

**TABLE 2:** Outcomes of endoscopic ultrasound‐guided tissue acquisition.

Needle gauge	
22 gauge, no. (%)	10 (62)
25 gauge, no. (%)	6 (38)
Needle type, no. (%)	
Expect SlimLine, no. (%)	11 (68)
SonoTip Top Gain, no. (%)	5 (32)
Number of punctures, median, (range)	2 (1–2)
Histologically or cytologically diagnosed malignant, no. (%)	16 (100)
Successful diagnosis of histological type, no. (%)	15 (94)
Immunohistochemistry, no. (%)	12 (75)
Final diagnosis of EUS‐TA	
Metastatic liver tumor, no. (%)	12 (65)
Hepatocellular carcinoma, no. (%)	2 (12)
Intrahepatic cholangiocarinoma, no. (%)	2 (12)
Primary site of metastatic liver tumors	
Colorectal cancer, no. (%)	5 (32)
Pancreatic cancer, no. (%)	3 (18)
Lung cancer, no. (%)	1 (6)
Breast cancer, no. (%)	1 (6)
Pancreatic neuroendocrine tumor, no. (%)	1 (6)
Not diagnostic, no. (%)	1 (6)^*^
Adverse events, no. (%)	0 (0)

Abbreviation: EUS‐TA, endoscopic ultrasound‐guided tissue acquisition.

* On the basis of the clinical course, this case was diagnosed as a metastatic liver tumor derived from pancreatic cancer.

In this study, there were no cases in which comprehensive genomic profiling (CGP) was performed with EUS‐TA specimens. Of the 34 primary malignant neoplasms (in 16 cases) in this study, 26 had already undergone resection. Therefore, when CGP is necessary, surgical specimens can be used, and EUS‐TA is mainly used to make histological diagnoses.

## DISCUSSION

Diagnosis becomes complicated when focal liver lesions appear in patients with a history of multiple primary malignancies. In such a case, diagnosis using imaging modalities and tumor markers has limitations, and histological evaluation is usually required. Previously, US‐guided or computed tomography‐guided percutaneous liver tumor biopsy was the first choice.

Meanwhile, EUS‐TA for focal liver lesions was first reported by Nguyen in 1999.^5^ Since then, reports of EUS‐TA for focal liver lesions have increased. Results were favorable, with 88%–100% sensitivity, 99%–100% specificity, and 0%–6% incidence of adverse events.^5^, ^8^, ^9^, ^10^, ^11^, ^12^, ^13^, ^14^, ^15^, ^16^, ^17^, ^18^, ^19^ EUS‐TA is particularly useful in cases in which percutaneous biopsy is difficult, such as small liver lesions, severe obesity, caudate lobe lesions, and massive ascites.^19^

EUS‐TA tends to use a thinner puncture needle (22–25 G) than percutaneous biopsy, with a small amount of tissue obtained initially. In early reports, cytological examination was performed as the diagnostic method.^5^ However, recent advances in puncture needles, called fine‐needle biopsy needles, have made the histological diagnosis combined with IHC possible.^13^, ^15^

Ichim et al. retrospectively studied 48 liver tumor cases in which EUS‐TA was performed and reported that the metastasis of various cancers, including gynecological tumors, could be diagnosed using IHC.^16^ However, the usefulness of EUS‐TA for local liver lesions in patients with a history of multiple malignant neoplasms has not been investigated.

In metastatic liver tumors with multiple primary malignancies, identifying the primary site is crucial. The treatment strategy differs depending on the primary site. The Japanese metastatic liver cancer clinical guidelines published in 2021 show the treatment strategy for each primary tumor in a flow chart.^20^ If the metastatic liver tumors are derived from ovarian cancer, gastroenteropancreatic neuroendocrine tumor (G1 and G2), colorectal cancer, or gastrointestinal stromal tumor, liver resection is the first choice. For metastatic liver tumors derived from gastric cancer, biliary tract cancer, pancreatic cancer, or breast cancer, chemotherapy is recommended. In patients with a history of multiple malignancies, the primary site must be histologically identified to provide the appropriate treatment. Histological diagnosis is also essential for determining chemotherapy regimens.

According to a previous study,^21^ 10% of focal liver lesions in patients with cancer are not metastatic; of note, primary malignant liver tumors and benign lesions are both considered differential diagnoses. In the current study, primary malignant liver tumors were observed in 25% (4/16), a frequency that cannot be ignored.

The complexity of diagnosing metastatic liver tumors varies depending on the histological types of the primary sites. When the histological types of the primary tumors are different (e.g., esophageal cancer: squamous cell carcinoma and colorectal cancer: adenocarcinoma), identifying the primary tumor is relatively easy, and diagnosis can be made using HE staining alone. Conversely, when the histological types of the primary tumors are the same (e.g., lung cancer: adenocarcinoma and colorectal cancer: adenocarcinoma), using IHC in combination is necessary.

Recent advances in pathological diagnosis using IHC have made the identification of the primary site of metastatic liver lesions possible in many cases with CK7 and CK20 staining and disease‐specific markers: hepatocyte, AFP (hepatocellular carcinoma), CDX‐2 (colorectal cancer), ER, PR (breast cancer), PSA (prostate cancer), and GATA‐3 (urothelial carcinoma).^22^, ^23^, ^24^, ^25^ In our study, IHC was performed in 75% of the patients and was useful in identifying the primary tumor.

The purpose of the biopsy must be considered when selecting a puncture needle. When biopsy is only for differentiating between benign and malignant lesions, a small‐diameter 25 G needle could be an option. For performing IHC or CGP, 22 G or 19 G needles should be considered. Ikeda et al. investigated the genomic profiling of EUS‐TA in 153 cases of unresectable pancreatic cancer and reported that the FNB needle and 19 G needle were independent factors of CGP analysis suitability.^26^ In cases requiring genomic profiling, EUS‐TA using a 19 G‐FNB needle is recommended.

Furthermore, when selecting a puncture needle, the maneuverability of the echoendoscope should be considered. When EUS‐TA is performed for the left lobe lesions transgastrically, the scope is easy to operate and a 19 G needle can be selected. In contrast, when performing a transduodenal biopsy for the right lobe, maneuverability may be poor, and 22 G or 25 G needles should be selected. If the biopsy is performed for the genomic profiling of right lobe lesions, US‐guided percutaneous biopsy using 16–20 G needles should be considered instead of solely relying on EUS‐TA.

The limitations of this study are that it is a single‐center, retrospective study, and the number of cases is small. In the future, it would be desirable to conduct clinical studies at multiple institutions with a large number of cases.

In conclusion, diagnosing focal liver lesions in patients with a history of multiple malignant neoplasms is of great clinical importance. In cases where the puncture is possible without intervening vessels (particularly left lobe lesions), EUS‐TA could be a useful diagnostic modality for focal liver lesions associated with multiple malignant neoplasms, allowing for the differential diagnosis of primary and metastatic tumors and the identification of the primary site of metastatic lesions.

## CONFLICT OF INTEREST STATEMENT

None.
